# Megafaunal Community Structure of Andaman Seamounts Including the Back-Arc Basin – A Quantitative Exploration from the Indian Ocean

**DOI:** 10.1371/journal.pone.0016162

**Published:** 2011-01-31

**Authors:** Sabyasachi Sautya, Baban Ingole, Durbar Ray, Sabine Stöhr, Kiranmai Samudrala, K. A. Kamesh Raju, Abhay Mudholkar

**Affiliations:** 1 National Institute of Oceanography (CSIR), Dona Paula, Goa, India; 2 Department of Invertebrate Zoology, Swedish Museum of Natural History, Stockholm, Sweden; University of Aberdeen, United Kingdom

## Abstract

Species rich benthic communities have been reported from some seamounts, predominantly from the Atlantic and Pacific Oceans, but the fauna and habitats on Indian Ocean seamounts are still poorly known. This study focuses on two seamounts, a submarine volcano (cratered seamount – CSM) and a non-volcano (SM2) in the Andaman Back–arc Basin (ABB), and the basin itself. The main purpose was to explore and generate regional biodiversity data from summit and flank (upper slope) of the Andaman seamounts for comparison with other seamounts worldwide. We also investigated how substratum types affect the megafaunal community structure along the ABB. Underwater video recordings from TeleVision guided Gripper (TVG) lowerings were used to describe the benthic community structure along the ABB and both seamounts. We found 13 varieties of substratum in the study area. The CSM has hard substratum, such as boulders and cobbles, whereas the SM2 was dominated by cobbles and fine sediment. The highest abundance of megabenthic communities was recorded on the flank of the CSM. Species richness and diversity were higher at the flank of the CSM than other are of ABB. Non-metric multi-dimensional scaling (nMDS) analysis of substratum types showed 50% similarity between the flanks of both seamounts, because both sites have a component of cobbles mixed with fine sediments in their substratum. Further, nMDS of faunal abundance revealed two groups, each restricted to one of the seamounts, suggesting faunal distinctness between them. The sessile fauna corals and poriferans showed a significant positive relation with cobbles and fine sediments substratum, while the mobile categories echinoderms and arthropods showed a significant positive relation with fine sediments only.

## Introduction

The exploration of the fauna associated with seamounts, underwater mountains of mostly volcanic origin, began over 50 years ago, after their initial discovery in the 1940s [1]. Originally, only structures of at least 1,000 m in height were included in the term seamount, but today the smallest topographic features termed seamount are merely 50–100 m in height [2]. Some studies have suggested that seamounts evolutionarily and ecologically ‘function as island groups’ [3], and potentially show a high degree of endemism. However, although different seamounts have been shown to harbor different and species rich faunas, observed endemism may be an artifact of undersampling [4], [5]. Few large studies that compare data from a wide range of habitats on seamounts and non-seamount areas have been conducted so far. For brittle stars, an abundant benthic group, O'Hara [6] found no difference in species richness and rates of endemism between seamounts and non-seamount areas in the Pacific Ocean. He found that, while seamounts vary in their faunal composition, in species richness and endemism, probably due to differences in their environment, as well as their geological and biological history, the same is true for the continental slope. The marine fauna in general is extremely undersampled, with an estimated 70–80% of marine species remaining to be discovered [7]. Thus, claims of endemism should be made with great caution, but the species rich environments on seamounts offer an opportunity to sample and study rare species with wide distributions. Since seamount summits are found at shallower depths than the surrounding bathyal sea floor, they are more accessible for research.

Seamounts function as hotspots for pelagic organisms, mainly fish, which has lead to overexploitation [8]. The benthic communities, which attract these fish, and the interactions between pelagic and benthic organisms, are little understood. An increase in knowledge on seamount ecology is thus vital for the management of a sustainable fishery and the protection of these vulnerable habitats.

Due to volcanic and hydrothermal processes, seamounts build up metal deposits that are potentially interesting for high-technology industries [9]. Large scale mining on seamounts may have severe consequences for the ecology of a whole region. Documentation of the faunal communities living on these seamounts, and the study of the biological processes controlling them are therefore essential for conservation.

Biological data are available for a small percentage of the ∼12,000 known seamounts [2], mainly from the Atlantic and Pacific Oceans. Of the Indian Ocean seamounts, 15 have been explored biologically, but only four are well documented with regard to benthic biology, from the others single records are known [10]. Particularly the seamounts of the Andaman Sea have not been explored before and are one of the geographic gaps that need to be filled, as recommended by Clark et al. [5]. In the present study we aim to explore the megafaunal community structure of the Andaman Sea seamounts using a quantitative approach.

Seamounts differ widely in environmental conditions and habitat properties, which is reflected in the observed differences in species richness and diversity [4], [5], [6], [11]. Among the important environmental factors, temperature is correlated to species richness, but to a lesser extent also depth, current velocity and other factors may affect the fauna on seamounts [11]. McClain [4] suggested that hard substrate seamounts have received considerably more attention than soft bottom ones, which may result in biased datasets. It is well known that hard bottoms promote the development of communities associated with sessile organisms, such as corals and sponges, whereas soft bottoms are typically inhabited by more or less motile fauna. Based on geomorphological features and geology, we aim to investigate how substratum types and shape affect the community structure within and between the seamounts. We assumed that hard substratum types such as boulders and cobbles will be species richer than fine sediment types on Andaman seamounts along with the basin region.

## Materials and Methods

### Study area

**Figure 1:**
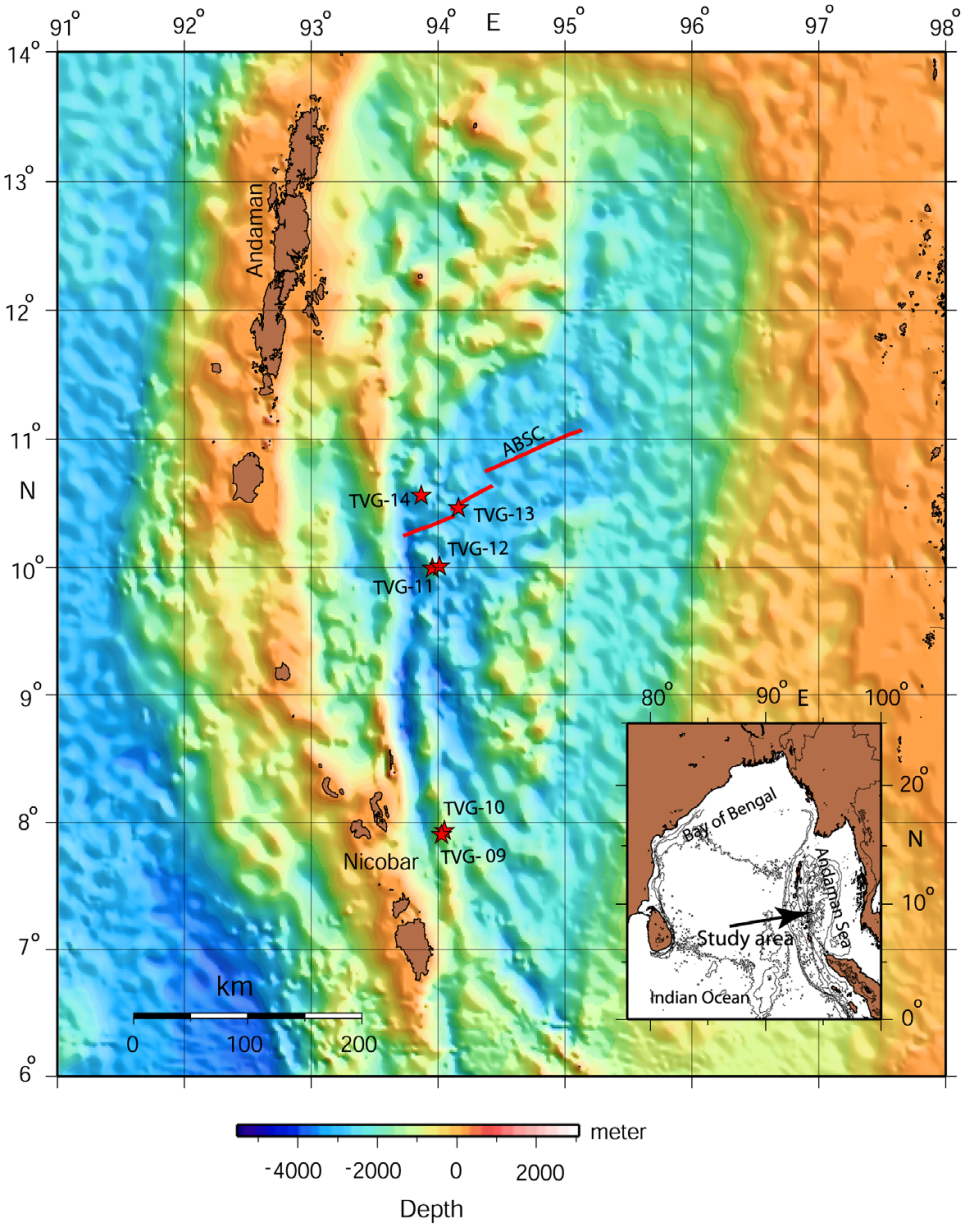
Bathymetric map of the Andaman Back-arc Basin including Andaman Back-arc Spreading Centre (ABSC) and locations of the underwater video transects (TVG). All the transects were located in the Back-arc Basin region only. The TVG-9 and TVG-10 were located on the cratered seamount (CSM) off Nicobar Island, TVG-11 and TVG-12 were located on the back-arc spreading ridge segments, while TVG-13 and TVG-14 were located on the rift valley of the basin floor and on the off-axial highs of the back-arc basin.

The volcanic-arc trench system of the Andaman Sea represents a submarine extreme boundary of the Indian plate in the northern Indian Ocean (Figure 1). The plate margins have several unique geophysical provinces, which include the arc-volcanoes, seamounts, deep sea valley, and the back-arc basin. The water depth varies from a few hundreds of meters to more than 3000 m, thus marking different ecological set-ups in the deep sea.

The Andaman Back-arc Basin (ABB) is an active marginal basin in the northeastern Indian Ocean, marking the eastern boundary of the Indian plate, sub-ducting beneath the Southeast Asian plate. Convergence of the plates leads to formation of several geomorphological features, like the Andaman-Nicobar island-arc, the Andaman back-arc spreading center, the seamount complex, and the back-arc basin [12], [13] (Figure 1). Recently a submarine volcano (crater seamount-CSM) with crater (160 m deep) on its summit was discovered in the Nicobar Earthquake Swarm area (07°55′N, 94°02′E) [14]. This seamount is conical in shape and has a steep slope, similar to aerial stratovolcanoes in the Andaman-Sumatra region. The SW-NE-trending, Andaman spreading center is characterized by a well defined rift valley in the NE part (maximum depth 3500 m) and is actively spreading at a spreading rate of 38 mm/year, this features bisects the basin [12]. Two underwater video surveys and sampling (TVG-9 and TVG-10) were carried out on the CSM off Nicobar Island. The flank of the CSM was surveyed during TVG-9 (average depth 594 m), while TVG-10 (average depth 434 m) was deployed inside the summit crater to investigate the crater-floor. Another seamount (SM2 at 10°N, 94°E, [12]), which is not a volcano but flat topped and part of the arc-parallel seamounts chain in the Andaman Sea, was explored with two more TVG operations and samplings. TVG-12 was deployed on the summit (average depth 1336 m) of the SM2, while TVG-11 (average depth 1357 m) was operated on the flank of this seamount. TVG-13 was deployed along the rift valley floor (average depth 2897 m) while TVG-14 was on the off-axial high (average depth 1791 m) of the Andaman spreading center.

### Data collection

Collection of megafaunal (video) data from six transects in the ABB was undertaken during November 2007 with the scientific research vessel *RV Sonne*. The details of sampling locations and depth are given in Table 1. Video sampling transects were selected using the EM120 multi-beam system data. We used the global seafloor topography from satellite altimetry and ship depth soundings data [15] for seamount mapping.

**Table 1:** Video observations on seamounts (CSM and SM2) and bathyal sea floor (back-arc basin) in the Andaman Sea.

Area	Station ID	Date	Start Long (E)	Start Lat (N)	End Long (E)	End Lat (N)	Min. Depth (m)	Max. depth (m)	Area covered (m^2^)
CSM	Summit (TVG-10)	26/11/2007	94°02.638	07°56.330	94°02.693	07°56.255	373	494	1242.4
	Flank (TVG-9)	25/11/2007	94°03.139	07°55.924	94°03.026	07°56.036	517	671	4159.3
SM2	Summit (TVG-12)	27/11/2007	94°00.784	10°00.243	94°00.813	10°00.255	1299	1372	972.3
	Flank (TVG-11)	27/11/2007	93°57.137	09°59.500	93°57.260	09°59.526	1290	1424	2484.8
Back-arc Basin	Off-axial highs (TVG-14)	30/11/2007	93°51.978	10°33.599	93°52.149	10°33.646	1767	1814	1404.4
	Rift Valley floor (TVG-13)	30/11/2007	94°09.500	10°27.200	94°09.590	10°27.590	2876	2917	1512.5

Detail of locations, depths and approximate area covered for each transect conducted by the TVG (television operated video gripper).

Seamount benthic communities were sampled using video transects collected with the TeleVision guided Gripper (TVG) system (0.6 m^3^). It was operated from the starboard side of the vessel. The system integrated 4 X ROS QL 3000 spotlight, 1XDSPLMSC 200 color, and 1XOSPREY OE 1390 black and white digital video telemetry systems, which provided a real time video link to the surface for maximum quality, digital through a fiber optical LWL cable. These video images were collected on DVD for further examination. The minimum length of the TVG tow was 389 m while the maximum was up to 1664 m. The vessel speed was approximately 0.7 knots during the TVG operations. The drop frame was towed in the water column between 1 and 3.5 m (dependent on bottom substratum types) above the seabed. The width of coverage of a single frame was approximately 2.5 m, but varied with distance from the bottom and angle of tilt. This width of the video has been used to calculate the total area covered by TVG during each transect sampling.

All megafauna was identified to lowest possible taxon during the underwater video observation or later by the taxonomists (see acknowledgement). Although every effort was made to identify the fauna to the lowest possible taxon, assignment to species was not always possible. For organisms that were morphologically distinct, but not sampled, and therefore not identified to species level, we used the higher taxon name with respect to different ‘Tag’ number such as Octocorallia sp.1, Octocorallia sp.2 or Actiniaria sp.1 etc. Several groups such as spider crabs, lithodid crabs, galatheidae, corals, sponges, feather stars, and sea stars, could not be confidently identified to species level from the video images and were grouped into larger categories. Other species were confidently identified to genus or higher taxon level. However, we collected some specimens using the hydraulic arms of the TVG for proper taxonomic identification. The locations were selected based on the video image and the TVG can be closed from onboard by transmitting a command to the arms. Samples were collected from the end point of each TVG location. Sessile fauna such as sponges and corals were carefully brushed from the rocks. Preliminarily, all collected fauna was preserved in 30% ethanol, but subsequently transferred to 70% ethanol. We also collected some bird nest sponges from the flank of the SM2 seamount, separated them carefully from the sediment and immediately preserved them in 70% ethanol. In the laboratory, all samples were washed carefully and the entire faunal community associated with sponge spicules was sorted out and preserved in 70% ethanol for further identification.

Videos were reviewed and megafaunal communities were identified and quantified. Bottom substratum types were determined by estimating the percentage of boulder, cobble, or fine sediment present, following the description of Hoff and Stevens [16]. The substratum types were classified by size, approximating the Wentworth grade scale [17], with boulders being defined as large rocks (>0.2 m diameter) to complete bedrock; cobble was rocks of 0.2–0.5 m diameter, and fines a bottom type of similarly sized gravel, sand and finer sediments of <0.05 m diameter. These groups were attributed an alphabetic code between #1 and #14 on overall composition of the bottom types (Table 2).

**Table 2:** Composition of substratum types used for assigning substrate codes to observed habitats viewed from video images from the TVG in the Andaman Back-arc Basin.

	% of each substrate type			
Substrate code	Boulder	Cobble	Fines	Substrate classification
#1	100			Boulder
#2	75	25		Boulder
#3	75		25	Boulder
#4	50	50		Boulder
#5	50	25	25	Boulder
#6	25	75		Cobble
#7	25	50	25	Cobble
#8		75	25	Cobble
#9		100		Cobble
#10	25	25	50	Fines
#11		50	50	Fines
#12	25		75	Fines
#13		25	75	Fines
#14			100	Fines

We defined each habitat patch as a video observation of about 5 minutes duration, which covered 108 m length. We used these habitat patches to calculate and quantify the substrate types and megafaunal abundances of each transect. The area of each habitat patch was determined by multiplying the transect width (average 2.5 m) with the length of the habitat patch. Then we compared the community composition within and between the seamounts.

Categories of motility were assigned to all observed taxa and analyzed to determine the functional role of dominant members of the seamount communities and the basin area. Organism motility was classified as sessile (e.g. sponges, corals), mobile (e.g. arthropods, chordates, ophiuroids, echinoids and holothuroids) and functional sessile (e.g. crinoids).

### Statistical analysis

Only those species that could be confidently identified from up to 3 m off the bottom were included in the analysis. The data were subjected to univariate analyses to study the benthic community structure, using Margalef's index [18] for species richness (d), Pielou's index [19] for species evenness (J'), and the Shannon-Wiener index [20] for species diversity (*H'* by using log_e_).

To investigate how similarity among assemblages changes with the substratum types and bathymetric gradients in the ABB, several multivariate analyses were conducted using routines in PRIMER v6 [21]. Following the general recommendations of Clarke and Warwick [22], the Bray-Curtis similarity measure was employed to assess multivariate similarity and dissimilarity between transects based on both presence/absence and log-transformed faunal abundance data. The significance of transects outlined a priori was tested with multivariate analysis (non-metric Multi-Dimensional Scaling (nMDS)) and the organisms which most contributed to the observed similarity within and dissimilarity among groups were found by means of SIMPER (similarity percentage). The habitat patches data of each transect were used for the nMDS analyses, using group averages, to explain the difference between transects for substrate types and megafaunal abundances. Using the RELATE test in PRIMER, we tested the Spearman rank correlation between the faunal similarity matrices as faunal abundance and percentage of groups, and model similarity matrix based on three different types of substratum, i.e. that faunal similarities are related to depth differences and substratum types among transects. Linear regression between biotic parameters (diversity indices) and environmental variables was tested using STATISTICA 6.

## Results

### Habitat structure

**Figure 2:**
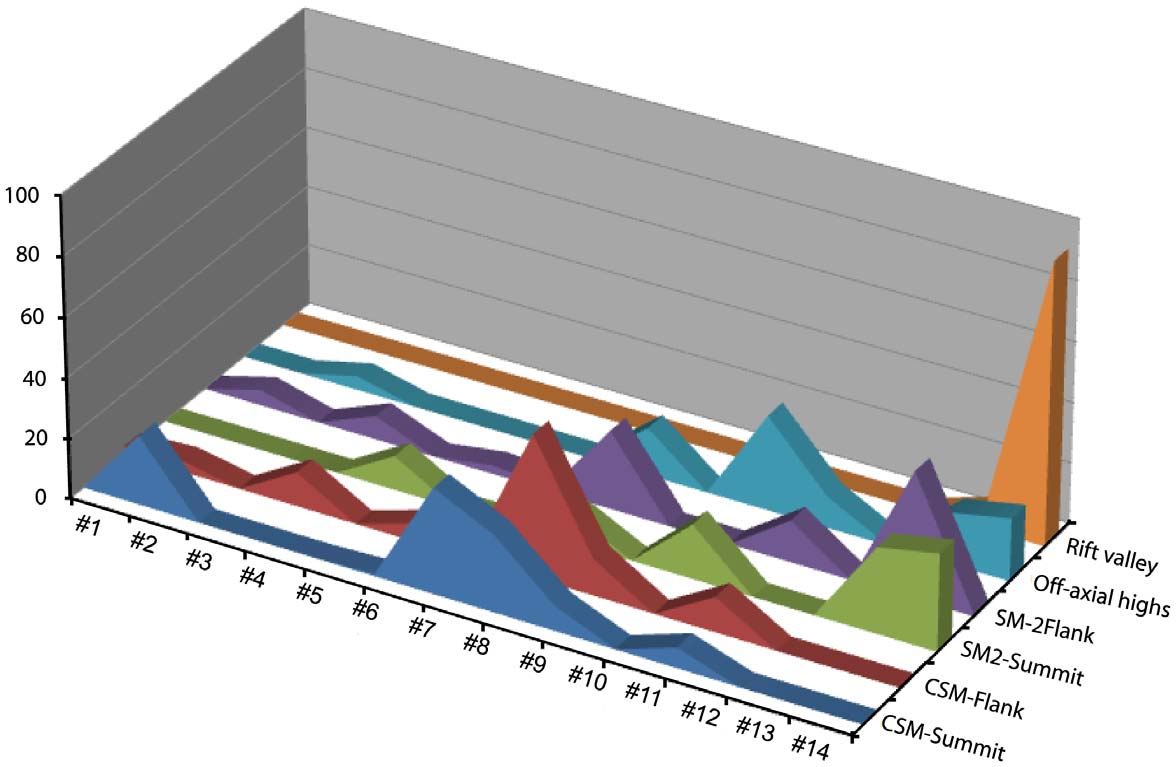
Composition of substratum types of two seamounts and surrounding sea floor in the Andaman Back-arc basin, with transect codes. Please see Table 2 for details of substratum types.

Diversity of substrate types was greatest for both seamounts, varying from boulders to fines (Figure 2). The boulders varied from smooth basalt spires to jagged uplifted slabs.

### CSM

Transects located at the CSM had mostly rocky substratum, while both transect, summit and flank were dominated by boulders and cobbles. However, eight unique combinations of physical substrate were observed from a total of 20 habitat patches at the CSM. The highest percentage of boulders (23.3%; code #2) and cobbles (47.8%; code #8) were recorded from the summit and flank of the CSM seamount. A lower percentage of fine sediment was observed at both transects.

### SM2

Ten combinations of physical substrate were observed on 13 habitat patches of the transects located on the SM2 (Figure 2). Fines and cobbles substrates dominated on both summit and flank. A maximum of 41.9% (code #13) and 32.3% (code #14) of fine sediments was observed at flank and summit respectively.

### Basin

Other transects (off-axial highs and rift valley) located in the back-arc basin, had a fine sediment type of substrates. Particularly, the rift valley had at maximum 93.8% fine sediments.

### Megafaunal community structure

A total of 948 individuals from 58 taxa, representing eight phyla, were observed in the collected samples and video images. The taxonomic catalogue is available online as supporting information (Figure S1).

**Figure 3:**
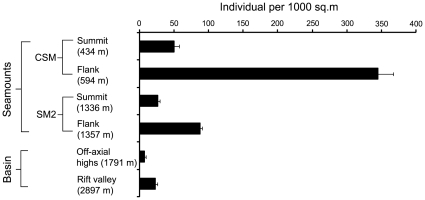
Megafaunal abundance along depth for two seamounts and the surrounding deep sea in the Andaman Back-arc basin.

Faunal abundances were highest on the seamount area and lower in the basin region (especially in deeper parts). Density counts varied from 7.4 to 345.2 ind. 1000 m^−2^ (mean 89.9±6.4) along the study area (Figure 3). The highest number of taxa (40) and individuals was observed on the flank of the CSM seamount (depth of 517–671 m).

Quantitative video transects at the CSM and SM2 seamounts differed in mean faunal density. Twenty habitat patches from two transects were identified between 373 and 671 m depth at the CSM seamount. These transect varied in length (497 to 1664 m) and width (1.25 to 5.8 m). Faunal density averaged 197.6±14.5 ind. 1000 m^−2^ (range  = 50 to 345.2 ind. 1000 m^−2^). Thirteen habitat patches from two transects were identified between 1290 and 1424 m at the SM2 seamount. These transect varied in length (389 to 994 m) and width (1.12 to 3.2 m). The faunal density averaged 56.8±2.9 ind. 1000 m^−2^ (range  = 25.9 to 87.7 ind. 1000 m^−2^).

### Assemblage composition and dominant taxa

**Figure 4:**
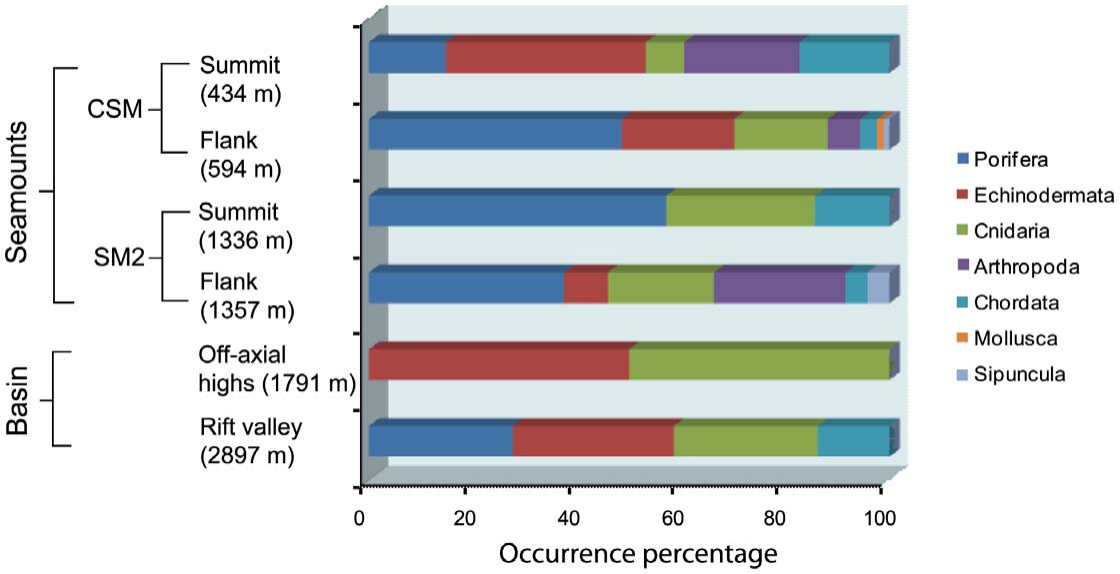
Megafaunal group composition along the CSM and SM2 seamounts and basin area in the Andaman Sea Back-arc Basin.

The overall megafauna was dominated by sponges (30.9%), but cnidarians (25.3%) and echinoderms (24.9%) were also important components along the study area (Figure 4).

### CSM

**Figure 5:**
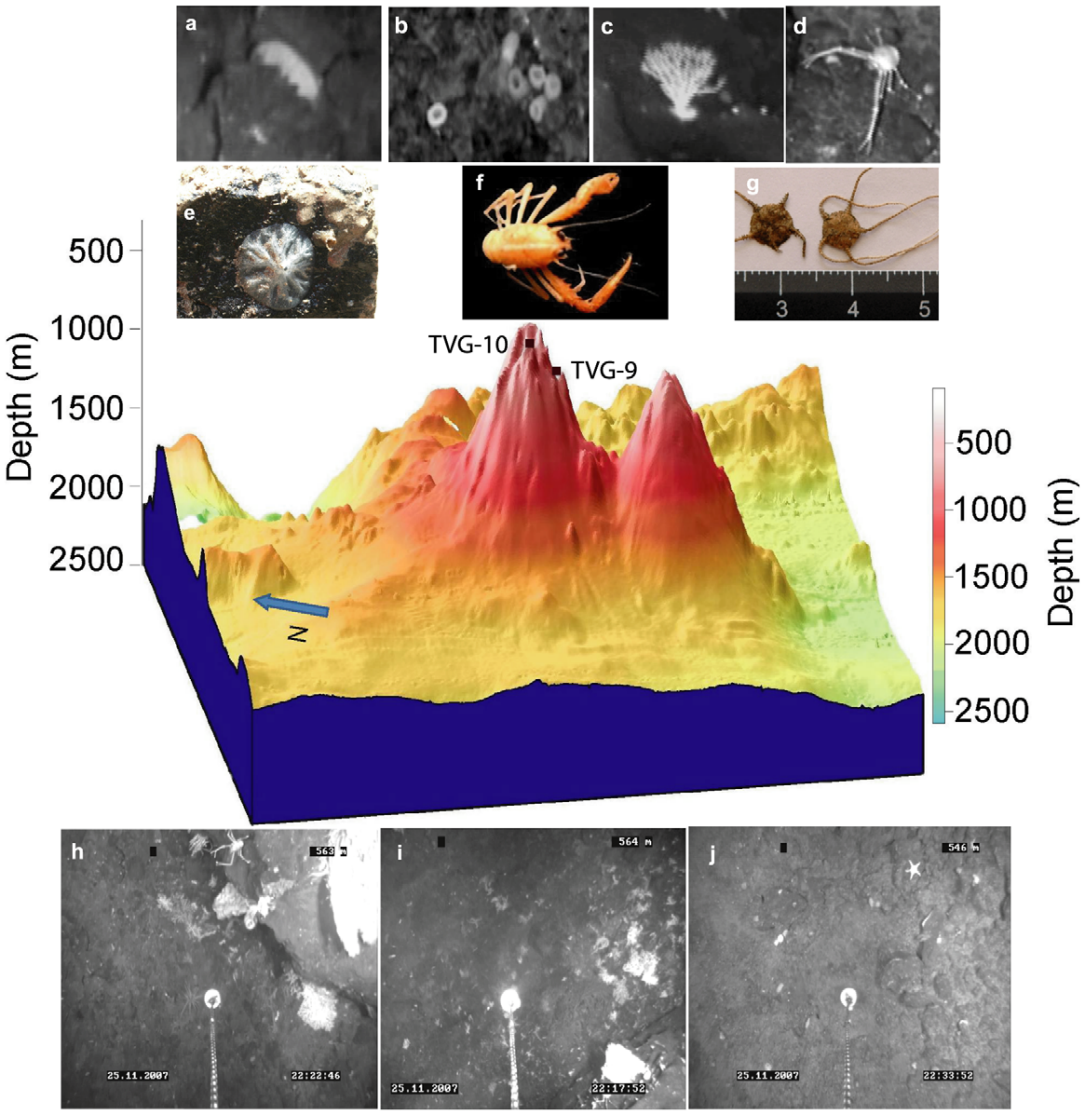
Structure of the CSM seamount (3D model midified from Kattoju et al. 2010) with accurate locations of the TVG-10 at summit and TVG-9 at flank, and the fauna associated with it. Megabenthic communities observed on a crater seamount in the Andaman Sea. a: Holothurid; b: *Euplectella* sp.; c: Gorgonian; d: Squat lobster - Galatheidae; e: Demospongiae attached to a big rock, onboard sample; f: the squat lobster-*Liogalathea laevirostris*; g: brittle star- *Ophiophyllum* sp; h: dense population of megafaunal communities (gorgonians, sponges, sea urchins, brittle stars, galatheids) lived on the big boulders and uplifted slabs; i: dense patches of corals (gorgonians); j: Ophiuroidea laid on the cobbles substratum.

The highest abundance (mean 345±21.5 ind. 1000 m^−2^) was observed at the flank of the CSM seamount, at depths between 517 m and 671 m, where the substratum was categorized mostly by cobbles mixed with fine sediments (code #8). Seven groups were found on the CSM seamount, all of them at the flank, while only five groups were represented at the flank. The sessile group porifera (48.6%) was clearly dominant on the flank, while the mobile groups echinoderms (38.4%) and arthropods (22.2) dominated at the summit (Figure 4). Among all the transects, the flank of the CSM exhibited the highest number (13) of cnidarians, although this group contributed to 18.0% of the total megafaunal abundance along the ABB. The hexactinellid sponge *Euplectella* sp. was well distributed and the most dominant taxon across the entire flank. It contributed with 33.6% to the total megabenthic community at the flank of the CSM, followed by demospongiae1 (10.5%) and *Corallium* sp. (3.2%), as the next dominant taxa in this region. *Ophiura* sp. (16.3%), demospongiae2 (14.8%) were the top ranked taxon at the summit of the CSM. The structure of CSM seamount and their associated megafauna is represented in Figure 5.

### SM2

**Figure 6:**
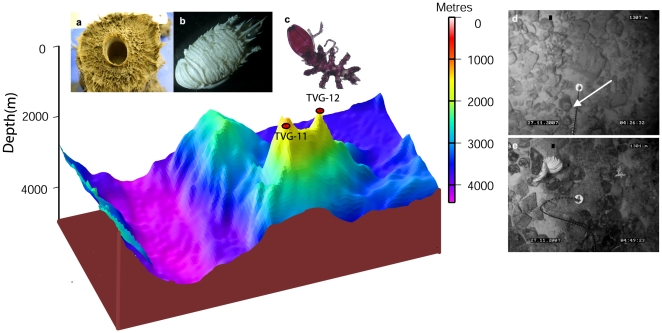
The SM2 seamount with locations of TVG-12 at summit and TVG-11 at flank, and its associated fauna. a:The bird-nest sponge *Pheronema* sp; b: *Munida* sp; c: Pycnogonid; d: Arrow indicating the underwater photograph of bird-nest sponge; e: Black coral attached to the hard substratum.

The second abundant (87.7±2.6 ind. 1000 m^−2^) area of the megafaunal community was observed at the flank of the SM2, at depths between 1299 m and 1424 m, where the substratum mainly consisted of fine sediments with cobbles (code #13). Six groups of megafauna were observed on the SM2, the flank exhibited all groups, while only three groups were found at the summit. The sessile group Porifera was dominant at both areas of the SM2, contributing 57.1% and 37.5% at the summit and flank respectively. Further, Arthropoda was the next dominant (25.3%) group at the flank of SM2, although this group was not observed at the summit. Cnidarians were the second dominant group next to the Arthropoda at the summit (28.6%) and flank (20.4%) respectively. The bird-nest sponge *Pheronema* sp. was the most dominant species on both areas of the seamount SM2 and contributed with 20.6% at the flank and 28.6% at the summit to the megabenthic community. Further, demospongiae3 and *Viminella* sp. were the next dominant at the flank of SM2 seamount. The structure of SM2 seamount is represented in Figure 6.

### Basin

Faunal abundances were poor on the basin transects. The observed abundance of megafauna on the transect on the rift valley was 23.1±2.2 ind. 1000 m^−2^ at depths between 2876 m and 2917 m, where fine sediments were completely dominant. Among four groups found in the basin area, all were observed on the rift valley, where off-axial highs showed two groups only. Echinoderms (especially holothuroidea5) and cnidarians (especially whip coral *Viminella* sp) were the dominant groups in the basin area. The most dominant group were the echinoderms (50%) at the off-axial highs, while they constituted 36% at the rift valley transect. The striking feature was that only the sponge *Hyalonema* sp. was observed on the rift valley located in the basin area.

**Figure 7:**
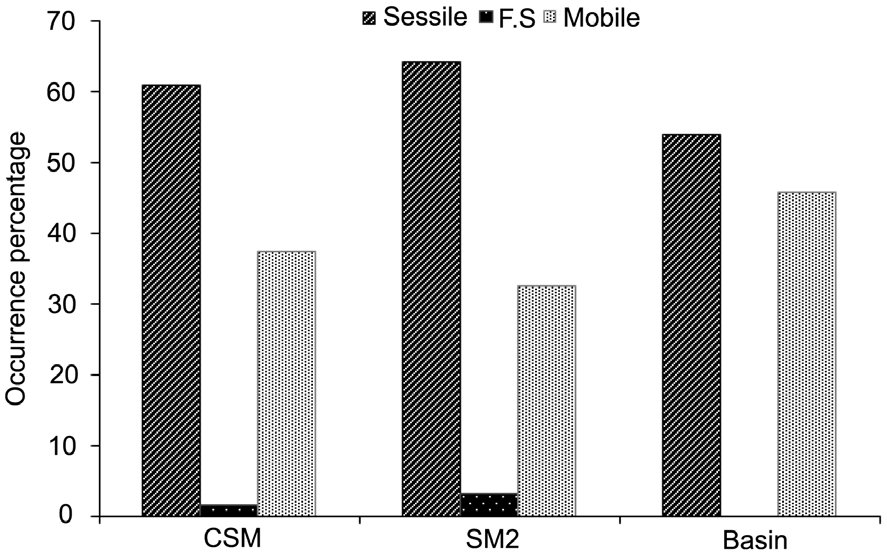
Occurrences of motility catagories on both seamounts and the basin area.

Sessile and mobile organisms accounted for most of the observations, while functionally sessile fauna was rare over the entire study area (Figure 7). Sessile fauna was the largest component of the community at the SM2 seamount, but the smallest in the basin area.

### Multivariate (MDS) analysis of substratum types and megafaunal community

**Figure 8:**
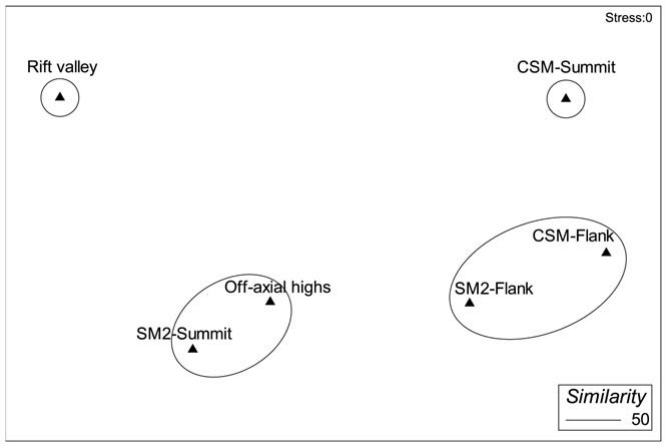
nMDS analysis of substratum types along the study sites in the Andaman Sea, the seamounts CSM and SM2, and the off axis basin.

The MDS plot based on the average percentage of substratum types found two groups with 50% similarity (Figure 8). Transects located on the flank of both seamounts formed group 1, because the substratum type cobbles mixed with fine sediments (code #8) contributed the highest similarity percentage (Table 3). Similarity of fine sediment types substratum allowed to form another group 2 between the summit of the SM2 and the off-axial highs in the basin area.

**Table 3:** SIMPER analysis of the substrate on two seamounts (CSM and SM2) in the Andman Back-arc Basin; average abundances (av. abund), average Simper (av. simp), contributed percentage (contrib%) and cumulative contribution (cum%).

	Codes	Av. Abund	Av. Sim	Contrib%	Cum%
Group 1 (flanks of CSM and SM2). Average similarity: 53.52	#8	36.73	26.06	48.69	48.69
	#11	12.71	11.62	21.71	70.4
	#4	9.61	7.88	14.73	85.13
	#2	4.49	3.72	6.96	92.09
Group 2 (SM2 summit and off-axial highs in basin). Average similarity: 65.63	#14	26.56	20.8	31.7	31.7
	#10	24.75	18.03	27.47	59.16
	#13	22.67	16.92	25.79	84.95
	#8	12.83	9.88	15.05	100

**Figure 9:**
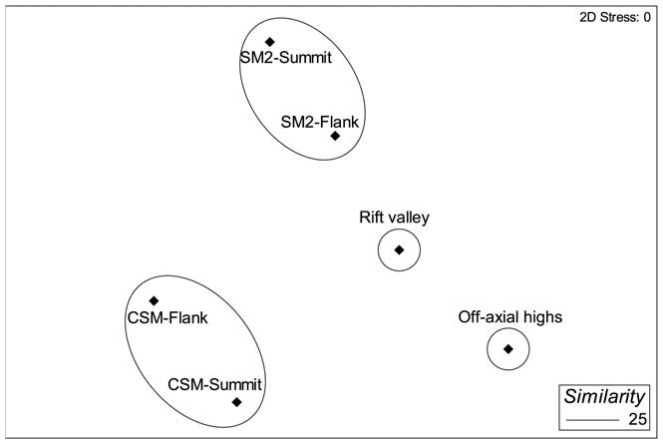
nMDS analysis of megafaunal community along the study sites in the Andaman Sea, the seamounts CSM and SM2, and the off axis basin.

The MDS plot based on the average abundance of megafauna also revealed two distinct groups with 25% similarity (Figure 9), each restricted to one of the two seamounts. Transects located on the CSM formed group A, while group B was formed by the SM2 transects. Some echinoderms (e.g., *Ophiura* sp., ophiuroidea1, holothuroidea1 etc.), asteroidea1, actinaria1, galatheidae and fishes played a major role in forming group A within the CSM transects (Table 4). In contrast, the bird nest-sponge *Pheronema* sp. and gorgonacea sp.2 were the most important organisms for forming group B on the SM2 seamount. The dissimilarity between the groups showed by the SIMPER analysis is presented in Table 5. Among 57 taxa observed on two seamounts, 46 taxa accounted for about 91% of the dissimilarity between the seamount faunal assemblages.

**Table 4:** Abundance SIMPER analysis of faunal communities on two seamounts (CSM and SM2) in the Andman Back-arc Basin; average abundance (avg. abund), average similarity (as. simp), contributed percentage (contr%), cumulative contribution (cum%).

	Species	Av. Abund	Av. Sim	Contrib%	Cum%
Group A (summit and flank of CSM). Average similarity: 26.15	*Ophiura* sp	1.88	3.27	12.5	12.5
	Ophiuroidea sp.1	1.93	3.27	12.5	25
	Asteroidea sp.1	1.61	3.27	12.5	37.5
	Holothuroidea sp.2	1.55	3.27	12.5	50
	Actiniaria sp.2	1.55	3.27	12.5	62.5
	Galatheidae sp.1	1.71	3.27	12.5	75
	Anguilliformes sp.1	1.66	3.27	12.5	87.5
	Actinoperygii sp.1	1.55	3.27	12.5	100
Group B (summit and flank of SM2). Average similarity: 26.56	*Pheronema* sp.	2.54	10.82	40.74	40.74
	Gorgonacea sp.2	1.64	7.87	29.63	70.37
	Elasmobranchii sp.1	1.55	7.87	29.63	100

**Table 5:** SIMPER analysis of average abundance dissimilarity between organism groups A and B on two Andaman Sea seamounts.

	Group A	Group B				
Species	Av.Abund	Av.Abund	Av.Diss	Diss/SD	Contrib%	Cum.%
*Pheronema* sp	0.77	2.54	3.91	1.14	4.26	4.26
Ophiuroidae sp.1	1.93	0	3.39	2.35	3.68	7.94
Anguilliformes sp.1	1.66	0	3.31	1.5	3.6	11.55
Galatheidae sp.1	1.71	0	3.16	1.92	3.44	14.98
Gorgoncea sp.2	0	1.64	3.12	1.74	3.4	18.38
Asteroidae sp.1	1.61	0	3.05	1.74	3.32	21.7
Holothuroidea sp.2	1.55	0	2.99	1.65	3.25	24.95
Actiniaria sp.2	1.55	0	2.99	1.65	3.25	28.2
Elasmobranchii sp.1	0	1.55	2.99	1.65	3.25	31.45
Actinopterygii sp.1	1.55	0	2.99	1.65	3.25	34.7
Demospongiae sp.2	1.06	0	2.98	0.81	3.25	37.94
*Ophiura* sp	1.88	0.77	2.75	0.79	2.99	40.93
Demospongiae sp.1	1.81	1.06	2.51	1.16	2.73	43.66
*Euplectella* sp	2.38	0	2.51	0.86	2.73	46.4
Decapoda sp. 1	0.77	0	2.17	0.81	2.36	48.76
Spider crab	0.77	0	2.17	0.81	2.36	51.12
Paragorgiidae sp.1	0.86	0.77	1.82	0.72	1.99	53.1

Average dissimilarity  = 91.92. Average abundance (av. abund), average dissimilarity (av. diss), quotient of dissimilarity and standard deviation (diss/SD), contributed percentage (contrib%), cumulative contribution (cum%). Here we presented the faunal contribution percentage for dissimilarity between the groups up to 50%.

### Diversity indices

**Figure 10:**
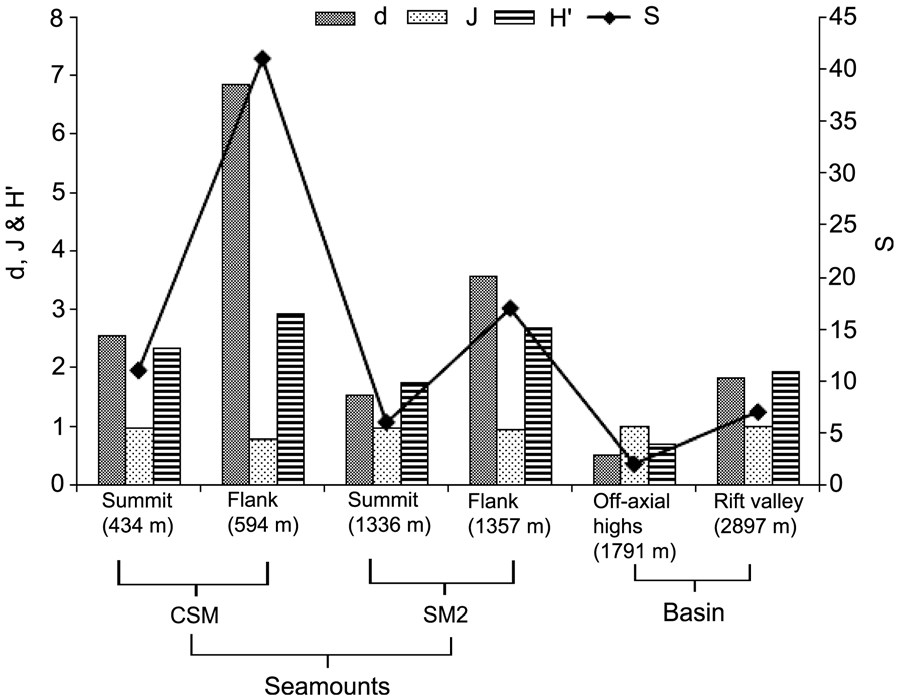
Transect-wise distribution of megafaunal community structure indices (S: number of species, d: Margalef's index, J': evenness, H': Shannon diversity).

The highest number of species (S) (41) was observed at the flank of the CSM, while the lowest (2) was recorded in the off-axial highs area (Figure 10). Margalef's index (d) of species richness varied from 0.5 to 6.8, the higher value recorded at the flank of CSM and the lower at the off-axial highs area. Pielou's index (J') of evenness varied from 0.8 to 1.0, with both transects on the flank showing lower values than other transects along the study area. Values of Shannon-Wiener index (*H'*) varied from 0.7 to 1.9 along the study area.

### Correlation between substratum types and biotic community parameters

Specific faunal groups exhibit varying responses related to substrate composition (Table 6). Total abundances (Log x+1 transformation) of the ABB showed a significant positive relationship with cobbles only. Porifera and Cnidaria exhibited the strongest positive relation with cobbles rather than with fines sediment, whereas Echinodermata and Arthropoda showed a significant positive relation only with fine sediments. No animal group showed a significant relation to boulders. The correlation between faunal diversity and substratum types was based on Pearson's correlation analyses (Table 7). Megafaunal species richness (S), Margalef's index (d) and Shannon-Wiener index (*H'*) were positively correlated with cobbles, while these three diversity parameters were negatively correlated with fine sediments. Again boulder did not play any significant role with relation to faunal diversity parameters. Moreover, substratum types did not show any correlation with motility categories.

**Table 6:** RELATE analysis between substratum types and biotic parameters in the Andaman Back-arc-Basin (ABB). Bold numbers indicate significant values.

Substrate type	Transformation	Group	Rho	ρ-value
Boulders	Log x+1	ABB total	−0.083	0.95
	P/A	ABB total	−0.073	1
		Porifera	−0.134	1
		Echinodermata	0.015	0.34
		Cnidaria	0.032	0.281
		Arthropoda	−0.043	0.71
		Chordata	−0.039	0.69
		Mollusca	0.024	0.29
		Sipuncula	−0.053	0.602
Cobbles	Log x+1	ABB total	**0.394**	**0.001**
	P/A	ABB total	0.024	0.39
		Porifera	**0.239**	**0.002**
		Echinodermata	0.093	0.062
		Cnidaria	**0.133**	**0.001**
		Arthropoda	0.055	0.151
		Chordata	0.024	0.247
		Mollusca	0.195	0.031
		Sipuncula	0.005	0.476
Fines	Log x+1	ABB total	0.302	0.00001
	P/A	ABB total	0.017	0.456
		Porifera	**0.143**	**0.007**
		Echinodermata	**0.118**	**0.013**
		Cnidaria	**0.115**	**0.02**
		Arthropoda	**0.122**	**0.037**
		Chordata	0.065	0.116
		Mollusca	0.079	0.157
		Sipuncula	−0.005	0.498

**Table 7:** Linear regression based on Pearson correlation showing the relationship between the substratum types and faunal diversity parameters in the Andaman Back-arc Basin.

Substratum type	S	D	J'	H'
Boulder	0.0376, p = .811	0.0321, p = .838	0.2645, p = .087	0.1622, p = .299
Cobbles	**0.6577, p = .000**	**0.4915, p = .001**	0.1285, p = .411	**0.4286, p = .004**
Fines	**0-.6065, p = .001**	**−0.4596, p = .002**	−0.2110, p = .174	**−0.4830, p = .001**

Bold numbers indicate significant values.

## Discussion

Seamounts are vulnerable environments and should be protected from destruction, which requires techniques and methods of documentation that cause as little damage as possible. To this end, most studies use under-water video images to explore the seamount fauna. McArthur et al. [23] suggested that fauna associated with hard substratum (e.g., cobbles, boulder) is best explored by underwater video or images. Accordingly, we have inventoried the megafauna of Indian Ocean seamounts, using under-water video.

The analysis of the video images showed that the volcano CSM consists of a rugged rocky environment made up of large boulder fields, and various sized cobble on the flank (Figure 5). Hard substrata, typical for the deep-sea environment, are common on seamounts and may take the form of rocks or cobbles [24]. Seamounts are primarily of volcanic origin, dominated by pillow lavas and basalts, which form boulders or cobbles later [25]. Accordingly, the CSM which has been reported as a submarine volcano, also presented the largest proportion of hard substrata, formed by boulders and cobbles. Underwater video analysis of substratum types also showed the highest percentage of boulders and lowest percentage of fine sediments at the crater summit of the CSM seamount. Further, this crater summit has been suspected as a mouth of the volcano, which may be the reason for the high percentage of boulders and cobbles on the CSM.

Compared to the surrounding areas of fine sediment-covered basin area, there was a marked difference in the abundance of coral and sponges on the seamount. The higher mean faunal density found on the CSM seamount, which has a shallower summit than the SM2 seamount, is likely due to nutrient availability, which increases with depth globally in concert with an exponential decline in faunal abundance and biomass [26]. In areas of upwelling, sessile suspension feeders, such as corals and sponges and the associated fauna, find suitable conditions on hard bottoms [16]. Hoff & Stevens [16] found for the Patton Seamount (Alaska) that suspension feeding communities were most abundant in the upper 1500 m, where it is still possible to take advantage of the photic zone. We observed lower faunal abundance and species diversity for the summit of the CSM seamount that is located just beneath the photic zone, than for the flank. Further, the SM2 also showed the same results as the CSM, with higher abundances and diversity on the flank than on the summit. This may be due to (unknown) differences in current velocities, because filter feeders require relatively fast currents [5].

The MDS analysis of faunal abundances did not find any group between seamounts, because of distinctness of the seamount faunal communities. This faunal distinctness between the seamounts was caused by those species which had their highest abundance on the same seamount and contributed some percentage to the dissimilarity (Table 5). Some species, such as *Euplectella* sp., were dominant and found only on the CSM seamount, whereas others, such as *Pheronema* sp., were dominant and only seen on the SM2. Dense aggregations of the bird-nest sponge are also known to occur at depths of 750–1300 m on the slope of the Porcupine Seabight in the NE Atlantic Ocean [27] and off Morocco [28]. Species presence restricted to a specific area is a character of endemism, although the degree of endemism cannot be ascertained from this study. Further, faunal distinctness between seamounts was also caused by differences in environmental conditions, such as depth and substratum types. During the underwater observation, it was found that faunal abundance changed with changing substratum types. This was confirmed by the MDS analysis, which showed that faunal abundance and substratum types followed the same pattern, and by the RELATE analysis, which showed a significant positive relation (ρ = 0.001) between abundance and substratum types (Table 5). Further, cobbles substratum also showed up in the MDS of substratum types, with 50% similarity between the flanks of both seamounts. Abundances were also higher on flanks than on summits of both seamounts and in the basin area. These findings support our hypothesis that geomorphology plays an important role for structuring the megafaunal communities in the ABB.

During the underwater observation we noticed that categories of motility changed with changing substratum types. However, faunal motility and substratum types did not show any correlation within the study area, probably because of the presence of some sessile categories such as sponges *Hyalonema* sp., Demospongiae sp.1 and whip coral *Viminella* sp., found attached to the hard substratum, as was occasionally observed in the basin transects. A large proportion of the fauna on the ABB seamounts consists of many attached and sessile, as well as mobile suspension feeders (sponges, corals, crinoids, brittle stars and holothuroids). The shallower transect, located at the summit crater, showed a comparatively large component of mobile *Ophiura* sp. It was observed that attached and sessile suspension feeders were fewer in the crater area than on the flank of the CSM seamount. This may be caused by to the geomorphological setting, such as the crater formation of the area, possibly creating a weak current flow, thus limiting the effect of the productive upwelling characteristics. Many sessile animals, such as gorgonians and black corals, require hard substrata and strong currents that supply them with food and oxygen, remove waste products and continuously keep the substratum, including the corals, completely clear of sediment [29], [30]. In the deep-sea such types of conditions are observed in very few habitats, seamounts are one of them. The high abundance and diversity of sessile fauna on both seamounts compared to the basin area also support our assumption that the hard substrata of seamount habitats are more favorable for rich megafaunal diversity than fine sediments.

### New insights from the present study

**Figure 11:**
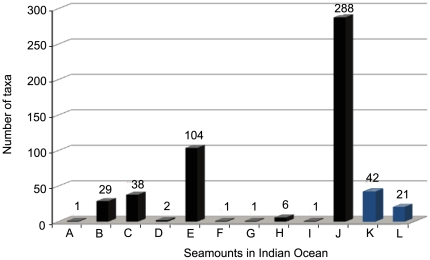
Number of taxa reported from the Indian Ocean seamounts including the present study. A: Bezrukov, B: Equator (Indian), C: Fred, D: Lena, E: Mount Error Guyot, F: Ob' Seamount, G:Shcherbakov, H: Travin Bank, I: Unnamed Seamounts – 1234, J: Walters Shoal, K: CSM - ABB, L: SM2- ABB.

**Figure 12:**
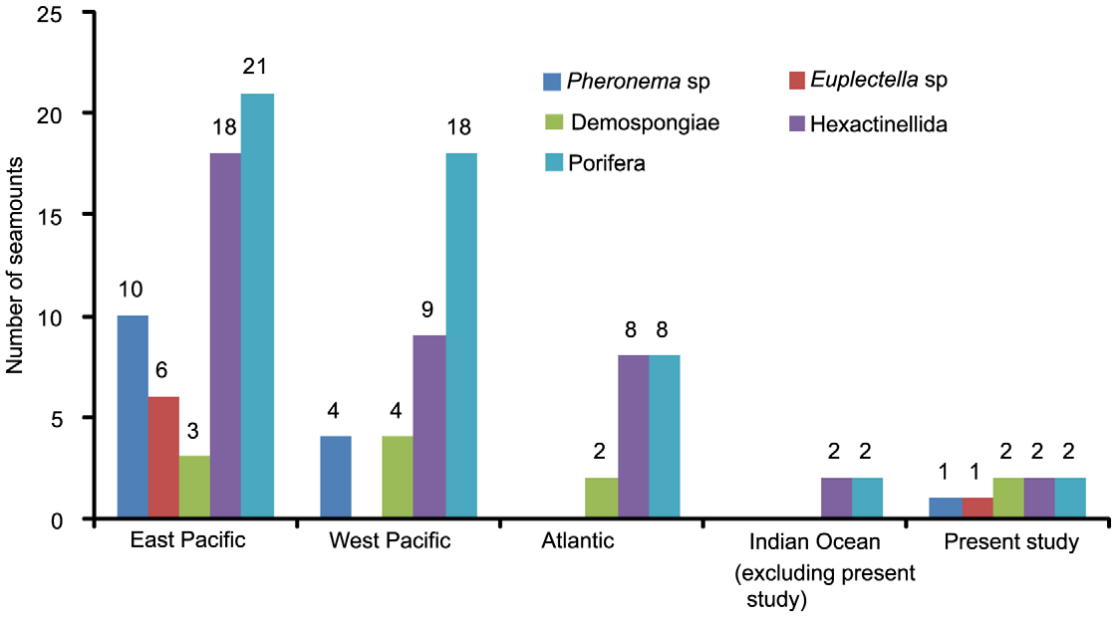
Comparison of sponges recorded from seamounts around the globe.

**Figure 13:**
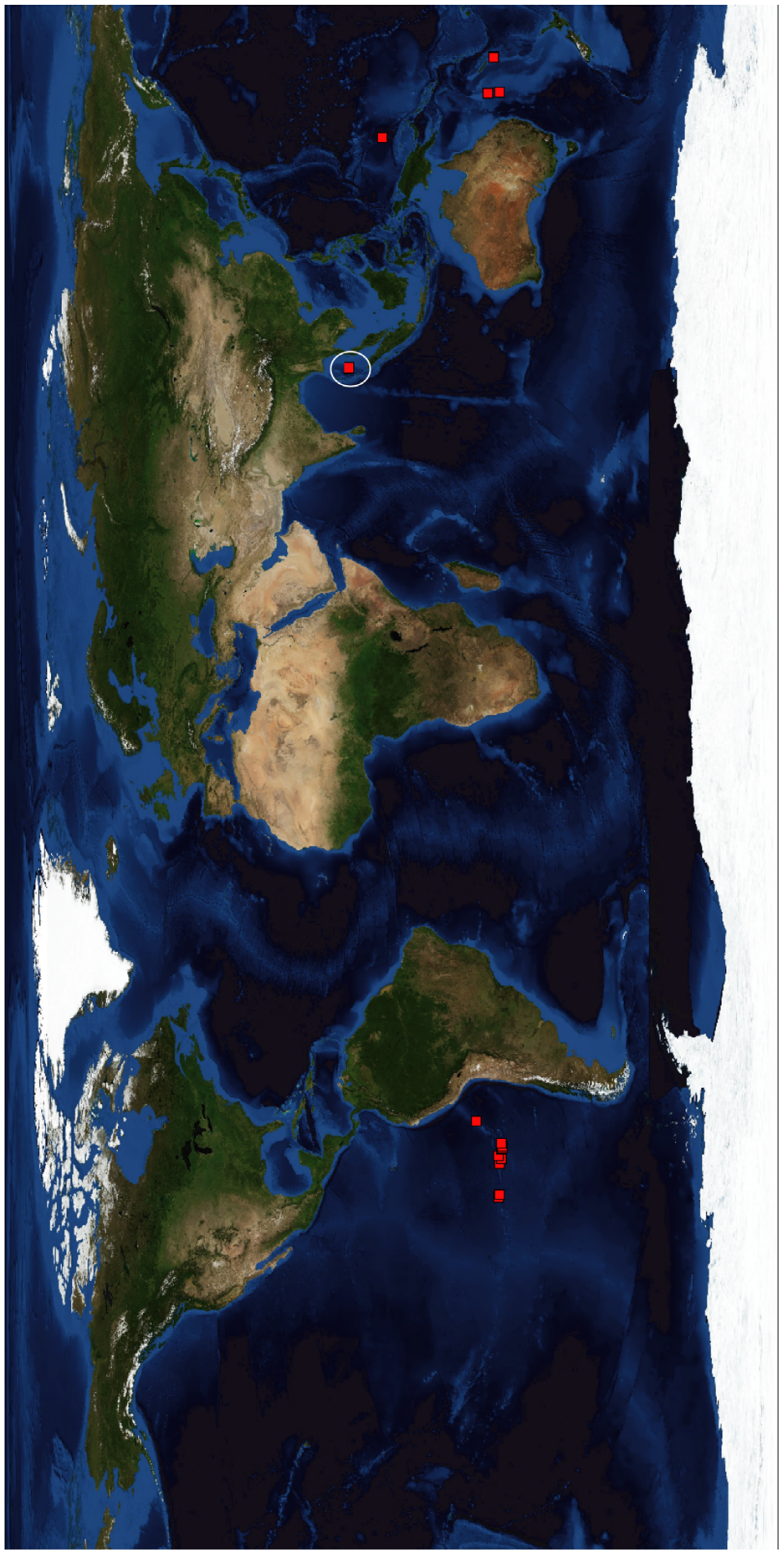
Map of *Pheronema* sp. distribution on the world ocean seamounts. The white circle indicates the new addition of *Pheronema* sp. from the seamounts in the Indian Ocean.

The degree of endemism and speciation on the Andaman seamounts is unknown, although new species (e.g., *Hyalascus andamanensis* Sautya et al., 2010 [31]) and new records (e.g., the epibiont *Thecacineta calyx* Schröder, 1907 [32]) have been reported from the CSM. Several of the ophiuroids collected by us are unknown species and will be described in a separate publication. Further, the present investigation provides additional knowledge of the seamount fauna as well as the deep-sea biodiversity of the Indian Ocean (Figure 11). Records of sponges from the seamounts of the world oceans (Figure 12) also confirm the earlier notion that the Indian Ocean is a poorly studied region. Prior to this study, there were only reports on Porifera and Hexactinellida from the Indian Ocean seamounts, while the present study not only added some more records of sponge species (e.g., *Pheronema* sp.; Figure 13) to the global seamount map, but also showed potential for the discovery of new species, if sampled systematically.

### Conservation of Andaman seamounts

The present investigation demonstrates the pristine condition of benthic communities on the seamounts, with negligible evidence of human impact. The communities are found in greater abundance and better health than those found in less-optimal habitat, suggesting that seamounts may be a source, rather than a sink, for some species [33]. Deep-water black corals (antipatharians) have substantial potential as proxy records of historical oceanographic and biogeochemical changes [34]. Their long life-span, wide geographic distribution and wider depth range [35] suggest that they may provide environmental information for geographic locations and for periods of time that are not available from other sources. Thus, they can be a potential source for paleoceanographic studies. Our study suggests that the seamounts in the Andaman Sea are biologically rich, home for many new species, and an optimal habitat for benthic organisms. We suggest that the region should be conserved for future biodiversity research. Conservation of these seamounts is also expected to ensure a survival and supply of ecologically important species that can disperse to depleted areas and replenish them.

### Summary and Conclusion

This study reveals several novel characteristics of the structure of megafaunal communities and their response to differences within and between the seamount habitats in the Andaman Back-arc Basin, Indian Ocean. The geomorphological settings, bathymetric gradient and substratum types in the study sites generate the habitat differences between the seamounts as follows:

1. CSM: This shallower seamount demonstrated a large component of hard substrates (e.g. boulders and cobbles), with higher species abundance and diversity. The sponge *Euplectella* sp. was dominant on the flank which was more diverse than the crater summit.
2. SM2: This seamount is characterized by cobbles and fine sediments types of substratum, with medium faunal abundance and diversity. The bird-nest sponge *Pheronema* sp. was dominant on the flank.
3. Basin: The basin area was dominated by fine sediments and very poor faunal abundance and diversity. Echinoderms were dominant in the basin area.

Faunal composition and diversity differed within and between the seamounts which can be explained by geomorphological features and substratum types. Due to lack of extensive sampling and other environmental data, this study was unable to explain the high species diversity on the shallower seamount. Further study is required to understand the processes involved in creating a high biodiversity, and other aspects, such as biogeography and endemism of megafaunal communities at different seamounts and also at other habitats like the ridge area in the Indian Ocean. Finally, it can be concluded that this study will be useful as a baseline for the Indian Ocean seamount region, which is still poorly investigated.

## Supporting Information

Figure S1 **List of megafaunal communities found along the Andaman Back-arc Basin.**
(RTF)
