# Supplementary material for: Megafaunal Community Structure of Andaman Seamounts Including the Back-Arc Basin – A Quantitative Exploration from the Indian Ocean
Source: PLoS One. 2011 Jan 31;6(1):e16162. doi: 10.1371/journal.pone.0016162 (PMC3031525; doi:10.1371/journal.pone.0016162)
Supplement: Figure S1 — List of megafaunal communities found along the Andaman Back-arc Basin. (RTF) [file pone.0016162.s001.rtf]

Taxa	CSM-Summit	CSM-Flank	SM2-Summit	SM2-Flank	Off-axial highs	Rift valley	
Euplectella sp	-	+	-	-	-	-	
Pheronema sp	-	+	+	+	-	-	
Ferrea sp	-	+	-	-	-	-	
Hyalascus andamanensis	-	+	-	-	-	-	
Hyalonema sp	-	-	-	-	-	+	
Aphrocallistes bocagei	-	+	-	-	-	-	
Demospongiae1	-	+	-	+	-	+	
Radiella sol	-	-	+	-	-	-	
Hexactinellida1	-	-	+	-	-	-	
Demospongiae 2	+	-	-	-	-	-	
Demospongiae 3	-	-	-	+	-	-	
Ophiophyllum sp1	-	+	-	-	-	-	
Ophiophyllum sp2	-	+	-	-	-	-	
Ophiolimna antarctica	-	+	-	-	-	-	
Ophiolepis sp	-	+	-	-	-	-	
Ophiura sp	+	+	-	+	-	-	
Ophiuroidea sp.1	+	+	-	-	-	+	
Asteroidea sp.1	+	+	-	-	+	-	
Holothuroidea sp.1	-	+	-	-	-	-	
Holothuroidea sp.2	+	+	-	-	-	-	
Holothuroidea sp.3	-	+	-	-	-	+	
Echinoidea	-	+	-	-	-	-	
Crinoidea sp.1	-	+	-	+	-	-	
Viminella sp	-	-	-	+	+	+	
Corallium sp	-	+	-	-	-	-	
Leiopathes sp	-	+	-	-	-	-	
Octocorallia sp.1	-	+	-	-	-	-	
Octocorallia sp.2	-	+	-	-	-	-	
Octocorallia sp.3	-	+	-	-	-	-	
Gorgonacea sp.1	-	-	-	+	-	-	
Gorgonacea sp.2	-	-	+	+	-	-	
Gorgonacea sp.3	-	+	-	-	-	-	
Paragorgiidae sp.1	-	+	+	-	-	-	
Antipathidae sp.1	-	+	-	+	-	-	
Actiniaria sp.1	-	+	-	-	-	-	
Actiniaria sp.2	+	+	-	-	-	+	
Actinoscyphia sp	-	+	-	-	-	-	
Pennatulacea sp.1	-	+	-	-	-	-	
Pennatulacea sp.2	-	+	-	-	-	-	
Nudibranch	-	+	-	-	-	-	
Chirostylidae sp.1	-	+	-	-	-	-	
Galatheidae sp.1	+	+	-	-	-	-	
Lithodidae sp.1	-	+	-	-	-	-	
Decapoda sp.1(Crab)	+	-	-	-	-	-	
Decapoda sp.2 (Spider crab)	+	-	-	-	-	-	
Decapoda sp.3 (Shrimp)	-	+	-	-	-	-	
Decapoda sp.4 (Shrimp)	-	+	-	-	-	-	
Decapoda sp.5 (Shrimp)	-	-	-	+	-	-	
Ammothella sp	-	-	-	+	-	-	
Liogalathea laevirostris	-	-	-	+	-	-	
Munida sp	-	-	-	+	-	-	
Valvifera	-	-	-	+	-	-	
Neotanaidae	-	-	-	+	-	-	
Anguilliformes	+	+	-	-	-	-	
Elasmobranchii 1	-	-	+	+	-	+	
Actinopterygii sp.1	+	+	-	-	-	-	
Actinopterygii sp.2	-	+	-	-	-	-	
Sipuncula	-	+	-	+	-	-	
